# Antimicrobial Resistance Analysis of *Escherichia coli* Isolated from Pigeons in Qingdao, Shandong Province, China

**DOI:** 10.3390/genes13091510

**Published:** 2022-08-24

**Authors:** Anqi Wang, Changmin Hu

**Affiliations:** College of Veterinary Medicine, Huazhong Agricultural University, Wuhan 430070, China

**Keywords:** pigeon, *Escherichia coli*, antimicrobial resistance, multi-drug resistance, genes, antibiotics

## Abstract

With the development of the pigeon industry in Qingdao, more attention is paid to scientific breeding and precise treatment. This study isolated and identified *Escherichia coli* from pigeons in Qingdao to determine their susceptibility to 18 antibiotics. The PCR method was used to detect the prevalence of Extended-Spectrum β-Lactamase genes, carbapenem resistance genes, 16S rRNA methylase genes and plasmid-mediated colistin resistance genes in the isolates. The results showed that *Escherichia coli* isolated from pigeons in Qingdao were the most resistant to tetracycline, followed by ampicillin, conventional cyclosporines, quinolones, cephalosporins, and aminoglycosides. No isolates were found to be resistant to amikacin, meropenem, colistin, tigecycline, and fosfomycin. The resistance to some antibiotics (ampicillin, tetracycline, and florfenicol) and the muti-drug resistance of *Escherichia coli* from meat pigeons were both higher than those from homing and ornamental pigeons. A total of 24.8% of the isolates showed multi-drug resistance, especially triple-drug resistance. Two isolates were found to carry ESBLs resistance genes. Hopefully, this study will provide a certain scientific basis for the clinical medication of pigeon colibacillosis, helping to prevent antimicrobial resistance transmission of *Escherichia coli* among different host animals and humans and maintain public health safety in Qingdao.

## 1. Introduction

Owing to geographical advantage and suitable climate, Qingdao has become one of the most suitable coastal cities for pigeon racing in China. With the improvement of people’s living standards, the demand for meat pigeons and ornamental pigeons is also increasing, and pigeon breeding has become an important industry in Qingdao. However, pigeons of all ages can suffer from some diseases resulting from pathogenic *Escherichia coli (E. coli)*, such as septicemia, air sac inflammation, and granuloma. It is difficult for pigeons to continue to survive after being infected with *E. coli*, and the dead pigeons cause the continued spread of *E. coli* disease [1]. Since the morbidity and mortality of the disease are relatively high, it poses a greater threat to the pigeon breeding industry and has aroused widespread concern in the society.

*Escherichia coli*, one of the normal flora members in the intestinal tract of humans and warm-blooded animals under normal circumstances, can be used as a test for direct or indirect fecal contamination in environmental hygiene and food hygiene and safety [2]. As an opportunistic pathogen, *E. coli* can widely exist in pigeon intestines, causing diarrhea, enteritis, and other diseases. Antibiotics have played a certain role in the treatment and prevention of *E. coli* infection, but the irrational use of antibiotics leads to the emergence and spread of antimicrobial resistance (AMR) of *Escherichia coli*, resulting in the increase in treatment costs [3], which has become a prominent problem in the pigeon breeding industry. Therefore, research on the antimicrobial resistance of *E. coli* isolated from pigeons is particularly important.

In recent years, there have been many studies on *E. coli* from pigeons. Xu, B. [4] reported the sensitivity of *Escherichia coli* isolated from pigeons to 20 commonly used antibiotics by the drug sensitivity test paper method, and found that it was moderately sensitive to gentamicin. Lv, M.N. [5] reported the AMR of *E. coli* and *Salmonella* from pigeons in Guangdong Province, China from 2011 to 2018, and found that the resistance of *E. coil* to different antibiotics was not the same in different years and the resistance to cephalosporins increased year by year. Wang, H.B. [6] reported on the drug sensitivity of *E. coli* taken from dead pigeons, detected by the conventional K-B drug sensitivity test paper diffusion method, and found that it was sensitive to ceftriaxone.

However, so far, there is no report on the AMR of *E. coli* from pigeons in Qingdao by the agar dilution method. As such, this research is aimed at Qingdao, using the above method to investigate the AMR of *E. coli* from pigeons and the prevalence of important antimicrobial resistance genes in order to provide a scientific basis for clinical antibiotics use in pigeon breeding, to prevent and reduce the harm of antimicrobial resistant isolates to pigeons and even to humans.

## 2. Materials and Methods

### 2.1. Sample Collection

In 2021, from July to August, the research was carried out in 3 homing pigeon breeding bases, 1 ornamental pigeon breeding base, and 3 meat pigeon markets in Qingdao (Shibei District, Chengyang District, Laoshan District, and Jimo District). Firstly, each sample pigeon was separated in a different cage for 7 days. Then, excreted fresh feces cotton swabs were collected from each pigeon aseptically to sterile containers at 4 °C and transferred immediately to the laboratory for analysis. Among them, there are 69 feces cotton swabs for homing pigeons, 50 for ornamental pigeons, and 30 for meat pigeons.

### 2.2. Isolation and Identification of Escherichia coli

The pigeon cotton swab samples were treated as follows: 100 μL of each fecal suspension was added to 1 mL of LB broth, shaken well, placed in a 37 °C incubator for overnight culture, and streaked on MacConkey agar. After culturing at 37 °C for 16–18 h, the morphological characteristics of the colonies were observed. Suspected colonies were picked and purified on MacConkey agar 2 to 3 times. One suspected bacterial isolate was selected from each sample for bacterial biochemical identification.

### 2.3. Antimicrobial Susceptibility Test

Minimum Inhibitory Concentrations (MICs) (µg/mL) were determined through the agar dilution method specified by the American Clinical and Laboratory Standards Institute (CLSI, 2015 [7]). *E. coli* ATCC 25922 was used as the quality control strain. The sensitivity of the *E. coli* isolates from pigeons to the 18 kinds of antibiotics (gentamicin, kanamycin, akamicin, spectinomycin, ampicillin, cefotaxime, ceftiofur, meropenem, nalidixic acid, levofloxacin, enrofloxacin, doxycycline, tetracycline, tigecycline, colistin, olaquindix, florfenicol and fosfomycin), belonging to 8 classes, was determined.

(1) Preparation of antibiotics: According to the solvent and diluent of each antibiotic in CLSI, the stock solutions of 18 kinds of antibiotics were prepared. The concentration of the stock solutions was 5120 μg/mL.

(2) Drug quality control: Using ATCC 25922 as the quality control strain, the effectiveness of the 18 kinds of antibiotics prepared was determined.

(3) Preparation of drug-containing medium: The antibiotics and their concentrations were marked on the disposable petri dishes, antibiotics were diluted by the doubling dilution method to make the final volume 1 mL; then,19 mL of MH agar were added (glucose 6-phosphate was added to the agar for the preparation of the fosfomycin drug plate) to the disposable petri dishes containing the antibiotic of each series of concentrations. These were Mixed well and air-dried, then a drug-free agar plate was prepared as a blank control.

(4) Inoculated bacterial solution: The *E. coli* and quality control bacteria were resurrected, 247.5 μL MH broth and 2.5 μL bacterial solution were added to each well of a 96-well plate, the bacterial solution was dipped with a sterilization stamp and printed on the drug-containing agar plate. When the bacteria were completely absorbed, they were put into a 37 °C incubator for 16–20 h’ culture.

(5) Interpretation results: The susceptibility of each tested isolate to 18 kinds of antibiotics was recorded and the AMR was interpreted according to the resistance clinical breakpoints in CLSI. The isolates that are resistant to three or more classes of antibiotics at the same time were called multi-drug resistance.

### 2.4. PCR Detection of Antimicrobial Resistance Genes

The DNA template was prepared by the boiling method. The primer sequences of the *bla_CTX-M-9G_* and *bla_CTX-M-1G_* encoding genes of Extended-Spectrum β-Lactamases (ESBLs) were synthesized according to the Liu [8] and Briñas [9] literature reports. The primer sequences of the 16S rRNA methylase genes *armA*, *rmtA*, *rmtB*, *rmtC*, and *rmtD* were synthesized according to the report of Doi [10]. The primer sequences of the carbapenemase-encoding genes *bla_NDM_* and *bla_KPC_* were synthesized according to the literature of Poirel [11]. According to the PCR reaction conditions of the above literature, single-plex PCR detection of antimicrobial resistance genes was carried out in a 15 μL PCR reaction system. The PCR system was as follows: distilled water 10.55 μL, PCR Buffer 1.5 μL, dNTP 1.2 μL, upstream and downstream primers 0.3 μL each, DNA template 1.0 μL, Taq 0.15 μL. According to the literature [12], the primers of the plasmid-mediated colistin resistance (*mcr*) genes *mcr-1*~*mcr-5* were synthesized and tested by multiplex PCR. Additionally the primers of *mcr-6*~*mcr-9* were synthesized for multiplex PCR detection [13]. The multiplex PCR system was: PCR Buffer 1.5 μL, dNTP 1.2 μL, upstream and downstream primers 0.3 μL each, DNA template 1.0 μL, Taq 0.15 μL, and 15 μL distilled water. After amplification, 1% agarose coagulation electrophoresis was performed. The positive PCR products were sent to MDBio Biotech Co., Ltd., Qingdao, China for sequencing (Table 1).

## 3. Results

### 3.1. Isolation and Identification Results of Bacteria

In 149 fecal samples randomly collected from 3 homing pigeon-breeding bases, 1 ornamental pigeon base, and 3 meat pigeon markets, a total of 149 isolates of *E. coli* were obtained through MacConkey medium screening and biochemical identification. Among them, there were 69 isolates from homing pigeons, 50 isolates from ornamental pigeons, and 30 isolates from meat pigeons. The samples’ separation rate was 100%.

### 3.2. Antimicrobial Susceptibility Test

**Figure 1:**
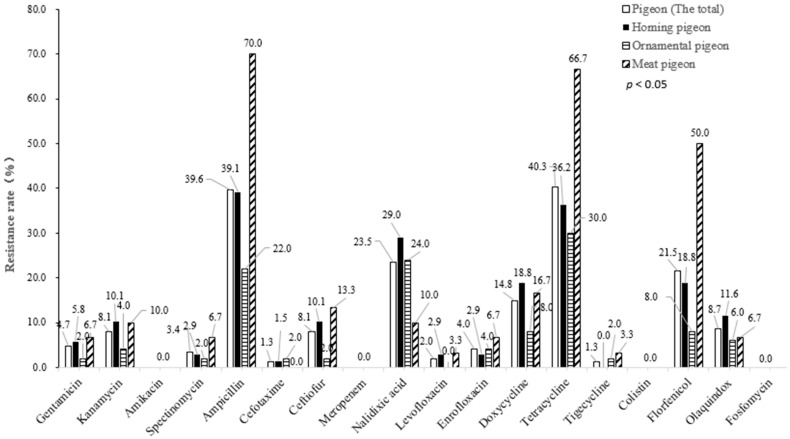
Antimicrobial resistance (AMR) of *Escherichia coli* (*E. coli*) isolates from different pigeon species to 18 kinds of antibiotics.

The antimicrobial susceptibility test showed that the 149 *E. coli* isolates were the most resistant to tetracycline, with a resistance of 40.3%, followed by ampicillin (39.6%). Among the quinolones, the resistance to nalidixic acid (23.5%) was the highest, followed by enrofloxacin (4.0%) and levofloxacin (2.0%). Among aminoglycosides, the resistance to kanamycin (8.1%) was the highest, and the rest were below 5.0%. The resistance to cephalosporins ranged from 1.3% to 8.1%. In addition, 21.5%, 14.8%, and 8.7% of isolates were resistant to florfenicol, doxycycline, and olaquindox, respectively. No isolates were found to be resistant to amikacin, meropenem, colistin, tigecycline, and fosfomycin. It is worth noting that the resistance of *E. coli* from meat pigeons to ampicillin (70.0%), tetracycline (66.7%), and florfenicol (50.0%) were significantly higher than that of those from homing pigeons to ampicillin (39.1%), tetracycline (36.2%), and florfenicol (18.8%). They were also significantly higher than those from ornamental pigeons to ampicillin (22.0%), tetracycline (30.0%), and florfenicol (8.0%) (*p* < 0.01). However, the resistance of *E. coli* from homing pigeons to nalidixic acid (29.0%) was significantly higher than that of those from meat pigeons (10.0%) (*p* < 0.05) (Figure 1).

**Figure 2:**
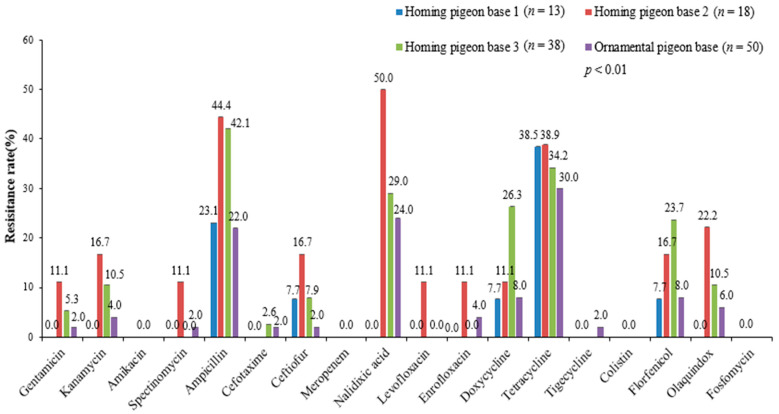
AMR comparison of *E. coli* isolates from homing and ornamental pigeons in different breeding bases.

**Figure 3:**
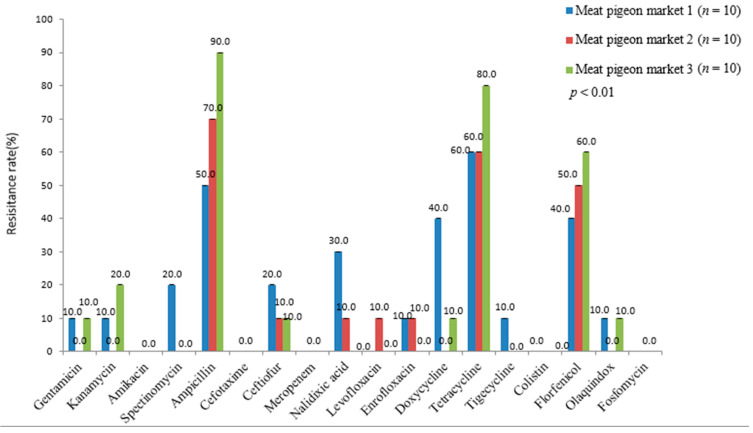
AMR comparison of *E. coli* isolates from meat pigeons in different breeding markets.

We compared the resistance of *E. coli* isolates from different bases/markets to the same antibiotic. We found that the resistances of *E. coli* from different homing-breeding bases to most antibiotics were different (Figure 2). For example, the resistance to florfenicol from the three pigeon-breeding bases were 7.7%, 16.7%, 23.7%, respectively. The resistance from base 2 to nalidixic acid (50.0%) was significantly higher than that from base 1 (0.0%). The resistance of *E. coli* from different meat pigeon markets to nalidixic acid, ampicillin, and doxycycline were also different (Figure 3).

### 3.3. Multi-Drug Resistance of Isolates

Among the 149 *E. coli* isolates from pigeons, 37 isolates (24.8%) showed multi-drug resistance, among which triple-drug resistance (12.1%) was dominant, followed by quadruple-drug resistance (6.7%). Four isolates showed resistance to six out of the eight classes of antibiotics tested. Among the 30 isolates from meat pigeons, 15 of them showed high multi-drug resistance (50.0%), which was significantly higher than that of those from homing pigeons (27.5%, 19/69) and ornamental pigeons (6.0%, 3/50) (*p* < 0.05). Additionally, the multi-drug resistance of the isolates from homing pigeons (27.5%, 19/69) was also significantly higher than that of those from ornamental pigeons (6.0%, 3/50) (*p* < 0.05). (Table 2)

### 3.4. PCR Test Results of Antimicrobial Resistance Genes

In the tested 149 *E. coli* isolates from pigeons, 16S rRNA methylase genes, the plasmid-mediated colistin resistance genes *mcr-1*~*mcr-9*, and the carbapenems resistance genes were not detected. Only the ESBLs resistance genes were detected in two isolates. Both genes belonged to *bla_CTX-M-1G_*. One was the *bla_CTX-M-3_* subtype, and the other was the *bla_CTX-M-15_* subtype (Table 3).

## 4. Discussion

In this study, 149 *E. coli* isolates from pigeons in Qingdao were obtained and the resistance of these isolates to 18 commonly used antibiotics was monitored. The findings promote the carrying out of discussions from the following aspects. First, the AMR comparison of *E. coli* between pigeons and other poultry in Qingdao could be achieved. This study showed that the isolates had the highest resistance to tetracycline (40.3%), followed by ampicillin (39.6%), conventional cyclosporines (14.8–40.3%), quinolones (2.0–23.5%), cephalosporins (1.3–8.1%), and aminoglycosides (0–8.1%). Liu, H.Y. [14] reported that the avian *E. coli* in Qingdao were resistant to ampicillin (97.2%), aminoglycosides (73.5–80.7%), quinolones (69.1–73.1%), and tetracyclines (71.1–87.2%); Wang, K. [15] reported that the resistance of *E. coli* isolates from chickens to tetracycline in Qingdao was 90%, and the resistance to gentamicin and cefotaxime was 88.7% and 87.1%, respectively. By comparison, we could find that *Escherichia coli* from pigeons was more sensitive to commonly used antibiotics than that from other poultry in the same area. The reason may be that husbandry practice in pigeons is better than for other birds, e.g., lower stocking density and a better rearing environment, resulting in the lesser occurrence of diseases. Therefore, lower selection pressure due to the relatively low usage of antimicrobials to control disease is achieved.

Next, the AMR analysis of *E. coli* from pigeons to the same antibiotic among different collecting places was obtained. By comparison, we found that the resistance of *E. coli* from different homing pigeon bases to the same antibiotic was quite different. The resistance of *E. coli* from different meat pigeon markets also had certain differences. The *E. coli* isolates from meat pigeon market 2 were sensitive to doxycycline, while the resistances of *E. coli* from meat pigeon markets 1 and 3 were 40% and 10%, respectively. *E. coli* isolates from homing pigeon breeding base 1 were sensitive to gentamicin, kanamycin, nalidixic acid, and olaquindox, while *E. coli* isolates from pigeon breeding bases 2 and 3 had different degrees of resistance to these antibiotics. It may result from different clinical medicines for pigeon colibacillosis in different pigeon markets or bases. Consequently, monitoring the antimicrobial resistance of *E. coli* from pigeons can help each base/market to formulate an antibiotic medication strategy that could meet its own needs.

Moreover, the AMR comparison of *E. coli* from pigeons in different regions was also realized. Lv, M.N. [5] reported that the *Escherichia coli* from pigeons in Guangdong Province, China were resistant to tetracycline (85%) and amikacin (50%). Wang, H.B. [6] reported that *Escherichia coli* isolates from pigeons in Changli, Hebei Province, China were resistant to spectinomycin, amikacin, and enrofloxacin. From this analysis, we could suppose that some factors, such as climate, environment, and the economy in different regions, lead to different selection pressures of *Escherichia coli* under antibiotics.

Besides, analysis of the resistance differences among different pigeon species could be implemented. In this study, the resistance of *E. coli* isolates from meat pigeons to ampicillin, tetracycline, and florfenicol reached 70.0%, 66.7%, and 50%, respectively, which was much higher than those from homing and ornamental pigeons. Additionally, it was found that the multi-drug resistance of the isolates from meat pigeons were also significantly higher than those from homing and ornamental pigeons. Bai, W.F. [16] reported that the resistances of *E. coli* from meat pigeons in Nanjing, China to ampicillin and tetracycline were both 85.7%; Xie, G.D. [17] reported that the resistances of *E. coli* from meat pigeons sold in Beijing, China to ampicillin and tetracycline were 90.4% and 97.1%, respectively. The results of this study are basically consistent with the reports in other areas mentioned above, indicating that the AMR of *E. coli* from meat pigeons in China is relatively serious. The reason may be that meat pigeons have a shorter breeding cycle, higher stocking density, and more extensive management system than homing and ornamental pigeons. For the use of fodder and antibiotics, there may be a lack of more complete and refined standards. Thence, the governmental supervision of the use of antibiotics in the breeding of meat pigeons needs to be strengthened.

In addition, the relationship of resistant *E. coli* between pigeon and other hosts in Qingdao was identified. Antimicrobial resistance can be transferred across bacteria via genetic elements, resulting in the rapid creation of multi-drug resistance in germs from animals [18]. In this study, two *E. coli* isolates were detected to carry *bla_CTX-M-1G_* genes (1.3%), which were sequenced as *bla_CTX-M-15_* and *bla_CTX-M-3_*, respectively. This was consistent with the findings of Qu, Z.N. [3], who reported that ESBL-producing *Escherichia coli* strains from chicken in Qingdao were also detected to carry *bla_CTX-M-15_* and *bla_CTX-M-3_*. The two *E. coli* isolates carrying *bla_CTX-M-1G_* in this study were resistant to cefotaxime, ceftiofur, and ampicillin, while sensitive to tigecycline, amikacin, and meropenem; which was basically equivalent to the antibiotic resistance pattern of *Escherichia coli* carrying *bla_CTX-M-1G_* from cattle in Qingdao reported by Lu, T.W. [19]. The above analysis suggests that there is the possibility of cross-transmission of resistant *E. coli* between different hosts in the same region. Various studies showed that run-off from manure application or livestock barns introduces unprocessed antimicrobial compounds and resistant organisms to soils and watercourses [20], and bioaerosols also influence the dispersal of resistant microorganisms through wildlife, domestic animals, soil, water, and humans [21]. Therefore, monitoring the AMR of *E. coli* from pigeons is necessary to avoid the further prevalence and spread of resistant *E. coli* among different hosts in the same region.

Furthermore, there is the possible transmission of resistant *E. coli* between animals and humans through various pathways, including direct contact, the food chain, or contact with animal excretion [22]. As the resident bird of Qingdao, the pigeon is linked closely to people’s lives. Drug-resistant *E. coli* remaining in meat pigeons may spread to humans through the food chain. The droppings produced in the public by homing and ornamental pigeons during flight may serve as vectors for the transmission of resistant *E. coli*. Through interviews with homing pigeon breeders, it is found that the flying distance of homing pigeons in Qingdao is mainly short or medium range (300–700 km), and homing pigeons hardly stop along the way during training and racing. Due to the droppings on the way, homing pigeons may increase the possibility of cross-infection of resistant *E. coli* between regions. Various studies revealed that pigeon feces in public areas represented a source of various zoonotic agents for humans and animals, and had been identified as a potential source and vector that could spread antibiotic-resistant bacteria and genes [23,24]. It seems that the gradual growth of AMR in pigeon may increase the risk of human infection, posing a certain threat to human health. Drug resistance increases the challenge around disease management, which has been documented to compromise the human immune system and increase complications and vulnerability after complicated surgeries involving cancer, knee replacement, dialysis, etc. [25]. Thus, monitoring drug-resistant *E. coli* from pigeons also takes great significance in maintaining public health safety in this region.

The development of AMR has been documented to be a natural process to enable survival of the bacteria, but has been fueled by human activities, including inappropriate prescription, excessive use of antibiotics as growth supplements in livestock, and the availability of few new antibiotics [26]. The mounting evidence around antibiotic usage practice being a crucial risk towards drug resistance necessitates the need for inculcating habitual and appropriately guided clinical management practices [25]. We should strengthen the feeding and management of pigeons, scientifically use veterinary antibiotics, reduce the emergence of numerous resistance mechanisms from overuse of antibiotics, and prevent its further spread to other host animals or humans.

Our study still has a limitation that molecular characterization of resistant isolates wasn’t carried out. The above resistance was analyzed according to international Clinical Laboratory Standard Institute (CLSI 2015 [7]) to describe the real AMR pattern in our study. Determining the genotypes of *E. coli* in pigeon to observe the potential mechanisms and characteristics of resistant strains should be our further study direction.

## 5. Conclusions

In this study, the antimicrobial resistance of *Escherichia coli* from pigeon in Qingdao was analyzed through the agar dilution method, and several common antimicrobial resistance genes in *E. coli* were investigated. The result showed that *E. coli* isolates from pigeons in Qingdao were the most resistant to tetracycline, followed by ampicillin, conventional cyclosporines, quinolones, cephalosporins, and aminoglycosides, while not resistant to amikacin, meropenem, colistin, tigecycline, and fosfomycin. The antimicrobial resistance of *Escherichia coli* from pigeons varies by region and species. The possibility of AMR transmission of *E. coli* among different hosts in the same region, even to humans, deserves our attention. The significance of this study is to call for the rational use of antibiotics to reduce the occurrence of drug-resistant *Escherichia coli*, prevent AMR transmission of *Escherichia coli* among different host animals and humans, and maintain public health safety in Qingdao. Additional research is needed to refine the results presented and to further investigate the effects on clinical medication and human life.

## Figures and Tables

**Table 1:** Primers for detection of genes.

Species, Gene, Primer		Sequence (5′–3′)	Amplicon Size, Base Pairs
Extended-Spectrum β-Lactamases (ESBLs) gene			
*bla_CTX-M-9G_*	*bla_CTX-M-9G_-F*	TGA CCG TAT TGG GAG TTT G	615
	*bla_CTX-M-9G_-R*	ACC AGT TAC AGC CCT TCG	
*bla_CTX-M-1G_*	*bla_CTX-M-1G_-F*	CTT CCA GAA TAA GGA ATC CC	765
	*bla_CTX-M-1G_-R*	CGT CTA AGG CGA TAA ACA AA	
16S rRNA methylase gene			
*armA*	*armA-F*	ATT CTG CCT ATC CTA ATT GG	315
	*armA-R*	ACC TAT ACT TTA TCG TCG TC	
*rmtA*	*rmtA-F*	CTA GCG TCC ATC CTT TCC TC	635
	*rmtA-R*	TTG CTT CCA TGC CCT TGC C	
*rmtB*	*rmtB-F*	GCT TTC TGC GGG CGA TGT AA	173
	*rmtB-R*	ATG CAA TGC CGC GCT CGT AT	
*rmtC*	*rmtC-F*	CGA AGA AGT AAC AGC CAA AG	711
	*rmtC-R*	ATC CCA ACA TCT CTC CCA CT	
*rmtD*	*rmtD-F*	CGG CAC GCG ATT GGG AAG C	401
	*rmtD-R*	CGG AAA CGA TGC GAC GAT	
carbapenemase-encoding gene			
*bla_NDM_*	*bla_NDM_-F*	GGT TTG GCG ATC TGG TTT TC	621
	*bla_NDM_-R*	CGG AAT GGC TCA TCA CGA TC	
*bla_KPC_*	*bla_KPC_-Fm*	CGT CTA GTT CTG CTG TCT TG	798
	*bla_KPC_-Rm*	CTT GTC ATC CTT GTT AGG CG	232
plasmid-mediated colistin resistance (*mcr*) gene			
*mcr-1*	*mcr-1-F*	AGT CCG TTT GTT CTT GTG GC	320
	*mcr-1-R*	AGA TCC TTG GTC TCG GCT TG	
*mcr-2*	*mcr-2-F*	CAA GTG TGT TGG TCG CAG TT	715
	*mcr-2-R*	TCT AGC CCG ACA AGC ATA CC	
*mcr-3*	*mcr-3-F*	AAA TAA AAA TTG TTC CGC TTA TG	929
	*mcr-3-R*	AAT GGA GAT CCC CGT TTT T	
*mcr-4*	*mcr-4-F*	TCA CTT TCA TCA CTG CGT TG	1116
	*mcr-4-R*	TTG GTC CAT GAC TAC CAA TG	
*mcr-5*	*mcr-5-F*	ATG CGG TTG TCT GCA TTT ATC	1644
	*mcr-5-R*	TCA TTG TGG TTG TCC TTT TCT G	

**Table 2:** The multi-drug resistance of *E. coli* from different pigeon species to 8 classes of antibiotics.

Species	Multiple Resistant Isolates (%)			
	Triple-Drug Resistance	Quadruple-Drug Resistance	Quintuple-Drug Resistance	Sixfold-Drug Resistance
Homing pigeon (*n* = 69)	8 (11.6)	5 (7.2)	3 (4.3)	3 (4.3)
Ornamental pigeon (*n* = 50)	1 (2.0)	1 (2.0)	1 (2.0)	0 (0.0)
Meat pigeon (*n* = 30)	9 (30.0)	4 (13.3)	1 (3.3)	1 (3.3)
The total (*n* = 149)	18 (12.1)	10 (6.7)	5 (3.4)	4 (2.7)

**Table 3:** Antimicrobial resistance genes in *E. coli* isolates from pigeon.

Antimicrobial Resistance Gene	Number of Isolates (%)
ESBLs genes	
*bla*_CTX-M-9G_	0 (0.0)
*bla*_CTX-M-1G_	2 (1.3)
16S rRNA methylase genes	
*armA*	0 (0.0)
*rmtA*	0 (0.0)
*rmtB*	0 (0.0)
*rmtC*	0 (0.0)
*rmtD*	0 (0.0)
carbapenemase-encoding genes	
*bla_NDM_*	0 (0.0)
*bla_KPC_*	0 (0.0)
*mcr* genes	
*mcr-1*~*mcr-5*	0 (0.0)
*mcr-6*~*mcr-9*	0 (0.0)

## Data Availability

Not applicable.
